# Vacuum Chamber Infusion for Fiber-Reinforced Composites

**DOI:** 10.3390/polym16192763

**Published:** 2024-09-30

**Authors:** Benjamin Grisin, Stefan Carosella, Peter Middendorf

**Affiliations:** Institute of Aircraft Design (IFB), University of Stuttgart, Pfaffenwaldring 31, 70569 Stuttgart, Germany

**Keywords:** fiber-reinforced composites, infusion, impregnation, VCI, vacuum chamber infusion, VAP, carbon fiber, thermoset, mechanical properties, void content, porosity

## Abstract

A new approach to an automatable fiber impregnation and consolidation process for the manufacturing of fiber-reinforced composite parts is presented in this article. Therefore, a vacuum chamber sealing machine classically used in food packaging is modified for this approach—Vacuum Chamber Infusion (VCI). Dry fiber placement (DFP) preforms, made from 30 k carbon fiber tape, with different layer amounts and fiber orientations, are infused with the VCI and with the state-of-the-art process—Vacuum Assisted Process (VAP)—as the reference. VCI uses a closed system that is evacuated once, while VAP uses a permanently evacuated open system. Since process management greatly influences material properties, the mechanical properties, void content, and fiber volume fraction (FVF) are analyzed. In addition, the study aims to identify how the complexity of a resin infusion process can be reduced, the automation potential can be increased, and the number of consumables can be reduced. Comparable material characteristics and a reduction in consumables, setup complexity, and manufacturing time by a factor of four could be approved for VCI. A void content of less than 2% is measured for both processes and an FVF of 39% for VCI and 45% for VAP is achieved.

## 1. Introduction

Fiber-reinforced plastic (FRP) composite materials are known for their combination of high mechanical properties, lightweight design flexibility, and non-corrosive properties and, hence, have been making inroads in aerospace, automotive, and trucking industries where fuel savings are very important [1]. The most commonly used fibers are synthetic fibers like carbon, glass, aramid, and natural fibers combined with different matrix systems such as thermoset or thermoplastic resin [2]. Each matrix system has advantages and disadvantages over the other. For thermoplastic material, better recyclability, higher production rates, and better impact behavior are stated [3]. Thermoset resins are mostly liquid at room temperature and curing due to an exothermal reaction [4]; therefore, the manufacturing processes can be handled without complex, heated consolidation tools, and the viscosities are lower by 3–4 orders of magnitude [5,6], making it possible to use infusion processes for impregnation [2]. The matrix properties regarding strength and stiffness are usually higher than they are for thermoplastic matrices, but they are more brittle [7]. The material properties can, hence, be adjusted due to the combination of fiber and the matrix system, where the orientation of the fibers has a major influence on the mechanical properties [8]. The manufacturing of semi-finished products as a basis for the preforming by the state of the art is performed on textile machines like weaving, braiding, or embroidery machines [2].

The choice of fiber, matrix, and manufacturing process highly determines the performance and field of application of a composite material [9]. Carbon fiber is used when high strength, high stiffness, or properties like minimum thermal elongation or electrical conductivity are essential. This includes applications such as aerospace components, measuring systems, and fast-moving machine parts with high positioning accuracy requirements. Glass fibers are usually used when the cost is a more dominant factor [10] and large amounts of fibers are needed that still offer good performance, like for the rotor blade wings of wind power plants, pipelines in the energy sector, or boat hulls.

The composite material, made of fiber and plastic, is created during the manufacturing process. As a result, different manufacturing methods, process variants, and fluctuations in the process parameters always lead to changes in the material properties, such as fiber volume fraction, void content, strength, and stiffness [11]. A major challenge in the production of FRP composites is the impregnation process of the reinforcement fibers with a liquid resin. Among several production techniques, Liquid Resin Infusion (LRI) methods like Seeman’s composite resin infusion process (SCRIMP) or vacuum-assisted process (VAP) are often used because they allow the reinforcement materials to be handled dry, have low investment costs, and can be adapted to manufacture a low-to-medium volume of parts [12]. In general, out-of-autoclave processes are becoming more relevant and show similar results to autoclave materials [13]. Among the many techniques included in LRI, VAP is a common one in the aerospace industry as it allows the production of high-quality composite parts with reasonable reproducibility. To reach the estimated quantities of future aircraft production [14], manufacturing processes must be improved by their sustainability to reduce their environmental impact, like waste reduction and, in parallel process, automation to enhance productivity. In particular, LRI processes are difficult to automate, since there is only a one-sided mold, and a complex set-up and integration of different process materials is needed [15]. Another drawback is the high number of consumables, which become waste after the process. The goal of this study is to verify a new approach that can improve these issues and have similar outcomes regarding the laminate quality. In addition, time-saving is aimed at. The new approach of the vacuum chamber infusion (VCI) offers the potential of a high degree of automation since the process is based on an existing, highly automated process known from the packaging industry. The VAP process has been selected as a reference since it is capable of producing minimum weight, high-performance laminates with properties comparable to those of laminates produced by more expensive processes such as autoclave cured prepreg, and close to what is expected for the VCI [16]. Since one goal is to improve the overall process efficiency, the material-efficient process DFP as a near-shape preform approach is used [17]. Additionally, DFP preforms have a low permeability in all three directions in-plane (K1 and K2) and out-of-plane (K3). The range is between 1 × 10^−15^ m^2^ and 5 × 10^−14^ m^2^, which is 1–2 orders of magnitude lower than it is for woven fabrics or non-crimp fabrics [18]. Subsequently, the infusion is challenging, but positive infusion results give a benchmark for other materials and allow a prediction about a feasible infusibility. The infusibility of the used DFP preforms by VAP has been approved and reported in a previous study [19].

### 1.1. Vacuum Assisted Process (VAP)—Infusion Processes for Composites

**Figure 1:**
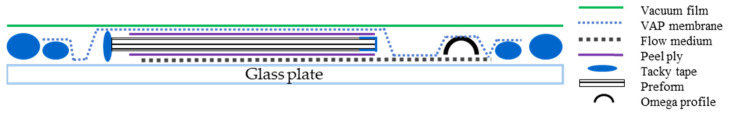
VAP infusion setup and principle.

The VAP infusion is a variation of resin infusion, where a semipermeable membrane separates the vacuum outlet from the surface of the part. This creates a full vacuum gradient and continued degassing across the part surface, as opposed to only at the end-edge of the part in traditional resin infusion like, e.g., SCRIMP. VAP and SCRIMP are used for the production of wind turbine blades, boats, aircraft interiors, and prototype parts. The main advantages of the VAP process are that all part sizes up to very large components with low permeable preforms, like, e.g., unidirectional carbon fiber spar cabs (>100 m), are feasible with very high process stability and low void content [20]. The main drawback is the high cost of the VAP membrane, which is not needed for SCRIMP. The difference between VAP and SCRIMP is that within the SCRIMP process, the air removal is only done on discrete lines or points, whereas in VAP the removal takes place extensively over the whole area of the part. All mentioned processes are well established and deliver high-quality laminates with high fiber volume content and low void content [21]. They have in common that the vacuum system is an open system with a permanent connection to the surrounding environment since the vacuum pumps are always running during the process. A sealed system is used for the VCI process, which is the main physical difference compared to VAP and SCRIMP. Figure 1 shows the VAP infusion setup and the principle, which has been used within this study. They have in common that the air outtake is done over the whole surface of the preform.

### 1.2. The Vacuum Packaging Process

Vacuum packing was invented by Henri de Poix of the Dewey Almy Chemical Company and patented in 1945. The process consists of three steps [22]:

1. A product is placed into a flexible and impermeable, plastic bag or container.
2. The atmosphere in the package is removed by evacuation.
3. The plastic bag or container is hermetically sealed to prevent transmission of moisture or gases into the sealed package.

The used bags usually consist of multilayer films, having different functions. The film packages and containers are mainly thermoplastic and are often thermoformed. The single layers of a multilayer film can fulfill different functions. One example is a multi-layer film consisting of PA/PE. The PA layer has a very low permeation to oxygen and moisture and guarantees that the vacuum is maintained, while the PE layer is used to enable the film to be welded securely. In addition, the PE layer can be embossed with a structure to create a flow aid for gases or liquids. The possibility of thermoforming specific packing materials like PETG opens the possibility of ensuring wrinkle-free and smooth mold surfaces, adding resin channels, and reservoirs for resin or vacuum reserve, and reducing material. The best case is to use a single-layer material because it is easy to recycle; a drawback of multi-layer film is that it is hardly recyclable [23]. The overall percentage of worldwide recycled thermoplastic material, which is counted into the annual global production of virgin material of 400.3 Mt (the year 2022), is low, at 8.9%. The percentage of used plastics in packaging in the EU is at 39.5% and, thus, accounts for the largest share of the total demand of 53.9 Mt [24].

### 1.3. Scope

The scope of this work is to analyze if similar results in mechanical properties and laminate quality of the VCI process in comparison to the state-of-the-art VAP process can be performed. Emphasis is put on the thermogravimetric and microscopic analysis of void content, fiber volume fraction, and the mechanical properties of tensile and bending strength and stiffness. Additionally, this study compares the economic aspects of the different processes and assesses possible fields of application.

## 2. Materials and Methods

### 2.1. Vacuum Chamber Infusion

The VCI process is a method to bring reinforcement fibers and liquid matrix together in a closed system with a flexible surrounding. The process is adapted from the vacuum packaging process. The fibers or the preform are put in a vacuum bag together with the matrix. Both components are positioned inside the bag in a way that they do not touch each other and that a complete evacuation of all free spaces is possible. The filled bag is then evacuated in a vacuum chamber and sealed. In the next step, the chamber is ventilated, and the impregnation process starts. Since a closed system is created, the process is going on until an equilibrium is reached.

**Figure 2:**
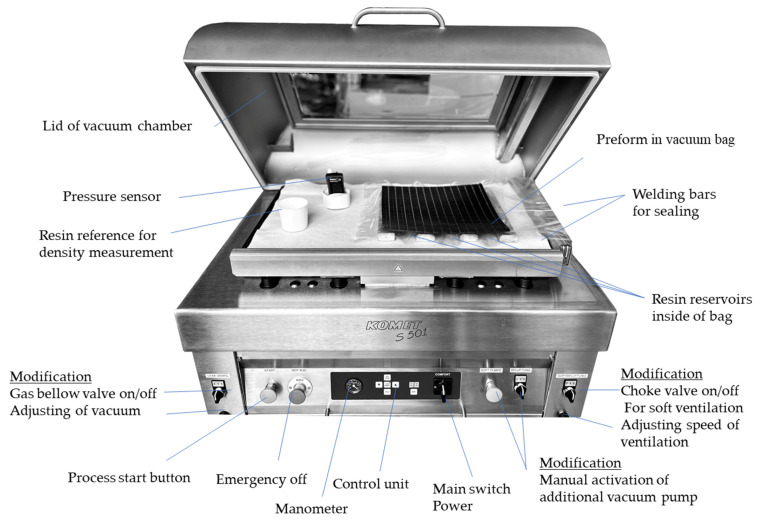
Modified Vacuum chamber machine for resin infusion, modifications are highlighted.

This study used a modified vacuum chamber sealing machine Komet S501 (Komet Maschinenfabrik GmbH, Plochingen, Germany), with a chamber dimension of 895 mm × 565 mm × 230 mm, for the infusion trials. The two welding bars have lengths of 740 mm and 455 mm. In Figure 2, the machine and the modifications are displayed, including the setup for the performed infiltrations of the sample plates. The machine includes two vacuum pumps for evacuation, one before the setup is sealed with a high flow rate and a second smaller one to adjust the vacuum inside the chamber after sealing if the chamber is not ventilated immediately.

To prevent the control from stepping into the sealing and ventilation sequence, a switch has been implemented to hold the vacuum. With this function, a pressure difference between the inside and outside of the bag can be adjusted between 0 and 1000 mbar. The machine has been equipped with a bellow valve to adjust the minimum pressure between 4 and 45 mbar. This is necessary to control the degassing or cocking of the resin during the process. The control panel allows to set a timer on how long the vacuum pump runs before the sealing and ventilation starts. The second function is the adjustment of the sealing time to adapt the sealing to the thickness and material of the vacuum bag.

**Figure 3:**
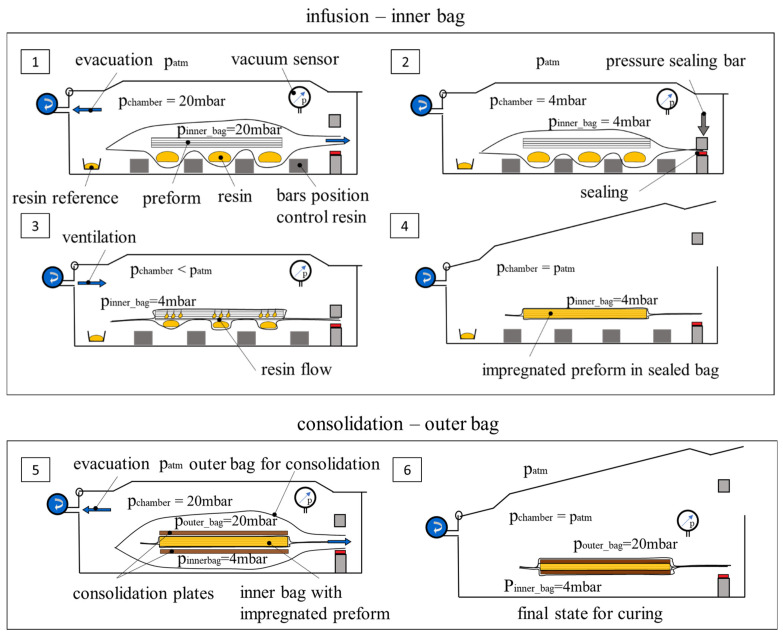
Scheme of the vacuum chamber infusion process. 1–4: Infusion with the inner bag. 5 and 6: Consolidation of the impregnated preform with an outer bag and 2 consolidation plates.

The VCI process parameters have been kept constant for all trials. For all trials, the resin has been prepared as follows. The resin component A is mixed with the degasser (1%-mass) and mixed by hand for 3 min. The mixture is degassed for 3 min/15 mbar. Afterwards, the hardener component B is added, and the mixture is stirred for 3 min. The last step is degassing for 3 min/15 mbar. The used vacuum bags are non-structured inside and consist of a two-layer film (PA6/PE). The outer PA6 layer is the gas barrier and the inner PE layer works as a sealing medium. The bags, type SR 400 × 600 PA/PE90 (Allfo GmbH & Co. KG, Waltenofen, Germany), have a size of 400 × 600 mm and a thickness of 90 µm. To measure the pressure inside the chamber, a vacuum measurement device is used, with a precision of ±1.0 mbar (Komet Maschinenfabrik GmbH, Plochingen, Germany, vacuum gauge with pressure equalization). Figure 3 shows the principle of how the infusion is performed. The numbering of the following description list is consistent with Figure 3; it describes the course of the process:

1. Setup of the inner bag for infusion
  - The resin is divided into three parts and poured into the bag separately
  - Four fixed bars beneath the bag prevent the three resin parts from distributing randomly
  - The preform is placed into the bag; it is carried by the bars and is not touching the resin
  - A cup with 20 g resin is placed additionally into the chamber as a reference for density measurement
  - The chamber lid is closed and evacuated, and pressure is set to 20 mbar
2. Evacuation
  - The pressure of 20 mbar is held for 3 min
  - The gas bellow valve is closed, and the pressure is decreasing
3. Sealing
  - When 4 mbar is reached, the bag is sealed
  - The ventilation is turned on and is slightly increasing the pressure inside
  - The impregnation by the resin flow immediately starts
4. Opening
  - The lid opens automatically when surrounding pressure ($p_{atm}$) is reached
  - The closed bag is taken out
5. Setup of the outer bag for consolidation
  - The inner bag, containing the impregnated preform, is straightened to remove folds, if there are any, placed between two stiff aluminum compression plates, and placed into the second outer bag
  - This setup is evacuated to 20 mbar and immediately sealed
6. Ventilation
  - The chamber is ventilated with maximum ventilation speed (the choke valve for soft ventilation is off)
  - The lid opens automatically when surrounding pressure ($p_{atm}$) is reached and the closed infusion and consolidation setup is taken out of the machine
    (1) $$p_{innerbag}<p_{outerbag}$$

A rule is set up for the pressure ratio of the inner and outer bag according to Equation (1). The inner bag has to have a lower pressure value than the outer consolidation bag. Otherwise, the inner bag will blow up during the evacuation and a distortion or wrinkling of the already impregnated preform can occur. In this study, a difference of 16 mbar has been chosen.

### 2.2. Materials

In this study, spread Mitsubishi 30k carbon fibers are processed into a one-sided bindered 20 mm wide tape on a spreading line (M&A Dieterle GmbH, Ottenbach, Germany, fixedTow production line) and placed via a 2D Dry Fiber Placement (DFP) machine (M&A Dieterle GmbH, Ottenbach, Germany, Crosslayer). All used materials are displayed in Table 1, showing the material properties of the fixedTow after spreading. The Mitsubishi 30k fiber is chosen for its good spreadability and consistent quality. The properties of the fixedTow and the raw fiber are displayed in Table 2 and Table 3.

### 2.3. Preforming: Tape Production and Dry Fiber Placement

To create the fixed tows, the fibers are spread to a width of 20 mm (±0.5) using a mechanical spreader and then secured in place with the binder. The used binder, in powdered form, is applied onto the spread tow and activated through infrared lamps. Subsequently, the tow is consolidated, cooled, and, ultimately, wound onto a film spool. The amount of binder utilized is consistent at 8% for all specimens, and activation of the binder occurs at 110 °C for all fixed tows.

To create the preforms, a DFP machine is used for the placement of the tapes. The fixed tows are placed at a laying speed of 8.5 m/min and an IR halogen lamp output of 360 W. A maximum of six plies were placed on top of each other. To produce preforms with more than six plies, the ply configuration is divided into several sub-preforms and stacked manually afterward. The preform size is 330 mm × 320 mm and the plies are placed on a plain weave glass fiber fabric, which serves as the placement substrate. The substrate only serves as a carrier for the placement process and is subsequently removed before the infusion. For adjacent plies with the same orientation, the layup path is parallelly shifted by 50% of the path distance. The path distance is set to 22 mm for all preforms. With this distance, a gap of 2 mm is implemented between each tape to lower the permeability compared to a layup with no gaps [4]. Table 4 shows the division of the subpreforms and how they are combined to create a symmetrical laminate.

### 2.4. Manufacturing of Plates, Sampling, Testing, and Analysis—VCI and VAP

For each sample configuration, the infiltration process, the fiber orientation, and the layer count are varied, as shown in Table 5. An amount of three plates each have been manufactured, a total amount of 12 plates. The size of the plates of 320 mm × 330 mm is chosen based on the required size of the samples for the mechanical testing, the chamber size, and the ease of handling of the preform and infusion set-up.

The last two columns show what kind of testing methods have been used to test the corresponding configuration mechanically. The curing has been performed for 24 h/RT. After removing the process materials, the plates have been post-cured for 6 h/80 °C in a convection oven. The resin reference samples for the determination of the density of the pure resin have undergone the same curing cycles. Each plate has its own batch of mixed resin.

**Figure 4:**
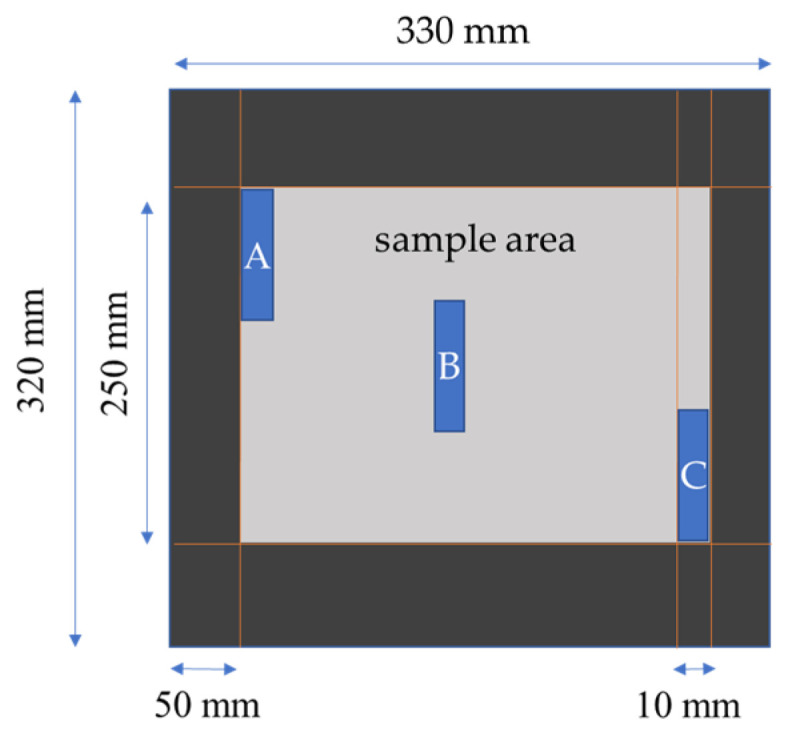
The geometry of sample plates. A, B, and C: areas for void content and fiber volume fraction. Grey area: area for tensile and bending specimens.

From each plate, nine specimens of 10 mm × 10 mm have been extracted at three different places (3 × A, 3 × B, 3 × C) to determine the fiber volume fraction, void content, and density (see Figure 4). Each plate has a resin reference sample from which the density has been determined (three specimens each) to get a higher accuracy for the void content analysis. The samples have been analyzed according to NORM EN 2564. From each of the 1 mm thick plates, six samples have been cut out with dimensions of 250 mm × 15 mm for unidirectional tensile testing. From the 2 mm plates, six samples of 250 mm × 15 mm have been cut for tensile testing, and additionally, six samples of 100 mm × 15 mm for bending tests. The bending test is performed with a Hegewald&Peschke “Inspekt Table 20” testing machine (Hegewald&Peschke Meß- und Prüftechnik GmbH, Nossen, Germnay) and a load cell of 20 kN according to DIN EN ISO 14125. The tensile test is performed with a Hegewald&Peschke “Inspekt Table 250” (Hegewald&Peschke Meß- und Prüftechnik GmbH, Nossen, Germnay) and a load cell of 250 kN, according to DIN-EN ISO 527-5 and DIN-EN ISO 527-4. The microscopic analysis is made with a digital microscope type Keyence VHX 7000 using an objective Keyence VH-Z250 series, type RZx250-x2500 (Keyence Deutschland GmbH, Neu-Isenburg, Germany). The areal void content is measured using the open-source software tool ImageJ (Wayne Rasband and contributors, Version 1.54g, National Institutes of Health, Bethesda, MD, USA) for digital image analysis. To achieve this, each image is calibrated using a scale derived from the Keyence microscope software.

## 3. Results

### 3.1. Fiber Volume Fraction, Void Content, and Density

In Table 6, all measured results for fiber volume fraction, void content, and density of the laminate, for the different configurations, are displayed. The values are the average values from all nine samples, which have been taken from each plate. For four plates, the average values of the void content are negative. Negative void content values are physically not plausible; they typically appear for the used method when the void content is very close to 0%. Since all plates have identical configurations concerning the preform and material, the values can be regarded as reliable relative to each other. Binders, especially, and other materials like sizing, can influence the curing of the resin and influence the density of the matrix. The resin reference sample for analyzing the matrix density of each resin batch did not contain any binder or fiber sizing. The density value of the fibers is taken from the supplier’s datasheet as suggested in the norm. In the technical datasheet, no deviation is given. The norm states, for its described method, “The absolute error in the determination of void content is estimated to be ±1%, due to uncertainties accumulated in the densities” [25].

**Figure 5:**
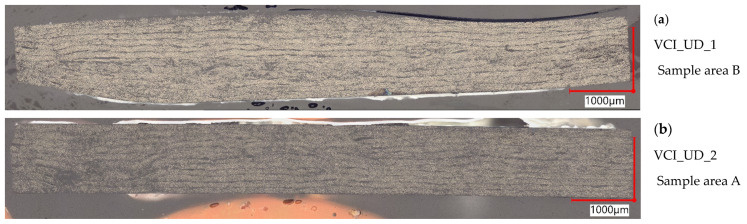

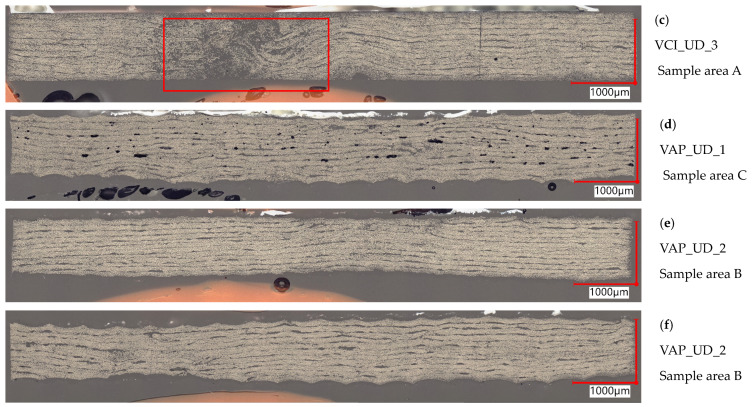
Representative cross-sections of the unidirectional configurations. Magnification: 250×, (**a**) VCI_UD_1 sample area B, (**b**) VCI_UD_2 sample area A, (**c**) VCI_UD_3 sample area A, (**d**) VAP_UD_1 sample area C, (**e**) VAP_UD_2 sample area B, (**f**) VAP_UD_2 sample area B.

**Figure 6:**
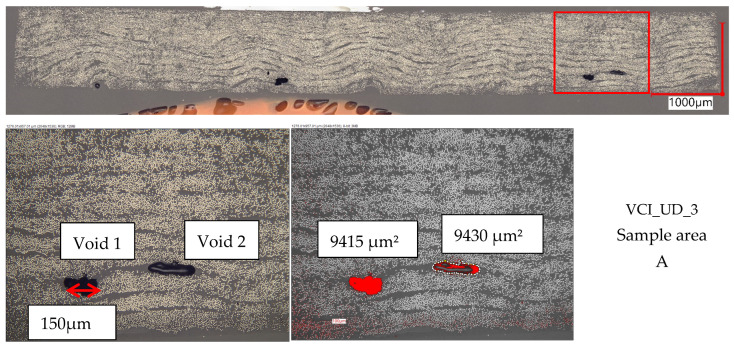
Representative cross-section with voids of the unidirectional configuration VCI. Magnification: 500×.

**Figure 7:**
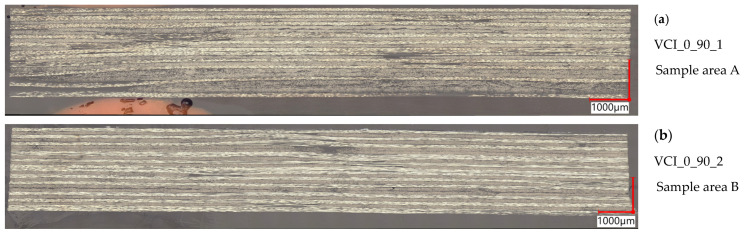

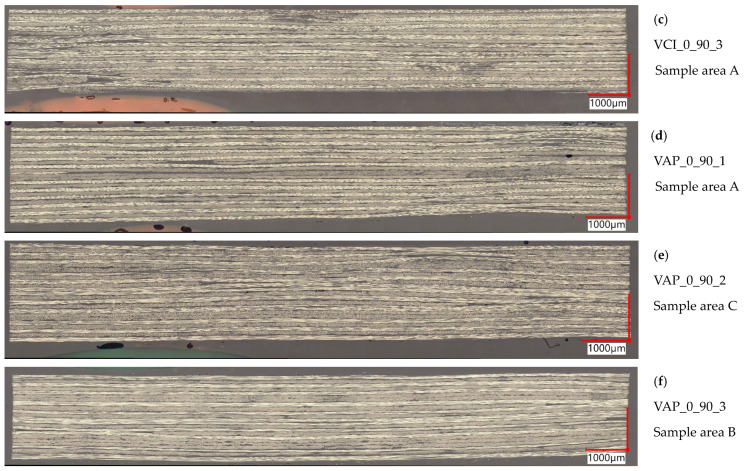
Representative cross-sections of the 0/90° configuration magnification 250×, (**a**) VCI_0_90_1 sample area A, (**b**) VCI_0_90_2 sample area B, (**c**) VCI_0_90_3 sample area A, (**d**) VAP_0_90_1 sample area A, (**e**) VAP_0_90_2 sample area C, (**f**) VAP_0_90_3 sample area B.

The average void content values are low for both processes: VCI is −0.02% and VAP is 1.41%, with the lowest single value for VCI at −0.66%. Taking an absolute error of ±1% into regard, the values are in the uncertainty range of the method. To prove that the void content is close to 0%, respectively, in the range of the measured values, cross-sections from all plates at all sample spots A, B, and C are taken. Figure 5, Figure 6 and Figure 7 display representative pictures from each configuration. The cross-sections show that for both process variations, only very few voids can be observed, which correlates with the measured void content values. Single samples show a local accumulation of voids. On average, the VCI process shows a lower absolute fiber volume fraction, with an average of 39.0%, compared to 45.2% for the VAP process. The deviation for VCI of ±2.7% is lower than that for VAP with ±3.7%. The densities of all laminates are in the range of 1.38–1.43 g/m^3^. The single values correlate with the corresponding FVF and void content values. The standard deviations are all in the typical range for the used method.

In Figure 5, representative pictures of the cross-sections from all unidirectional plates are displayed. The unidirectional VCI plates partially show a high undulation in 90° direction locally, thus introducing resin-rich zones. The effect can also be observed on the plate surfaces directly. The effect appears when the inner bag is not properly stretched before putting the setup in the outer bag for consolidation. This effect is not observed for the unidirectional plates manufactured with VAP since wrinkles in the membrane and vacuum bag can be handled when the vacuum is applied, before opening the resin intake. When having a stretched inner bag, the VCI plates show a homogeneous thickness distribution (see Figure 5b,c); the thickness distribution is highly varying when stretching is not ensured (see Figure 5a). On the unidirectional VCI samples, a local fiber-washing effect can be observed. The resin content is high, and the fibers are not in the layer place anymore (see Figure 5c). The fiber distribution at these spots leads to the assumption that a resin channel is formed during the infusion and the fibers are pushed out of place vertically and horizontally. The spots look like a flow vortex. The unidirectional VAP plates show an in-homogeneous thickness distribution over all three plates, which can be seen in Figure 5d–f. A regular waveform is observed on both surfaces of the samples, which introduced undulation in the surface layer in 90° direction. The form can be introduced by the peel ply or the flow medium.

All pictures show a low void content. Only single voids are overserved locally within all 18 analyzed cross-sections of the unidirectional plates. This finding correlates with the measured void contents. An exception is plate VAP_UD_1. It seems that during the production of the plate, VAP_UD_1 one or several process parameters have not been correct. The measured void content of 2.89% is higher than for all other plates and the only one greater than 2%. The cross-sections of all sample areas, A–C, show a clear difference in the void content. For comparison of overserved effects between the processes and findings, this plate is spared out.

For one representative sample, a reference measurement is performed on the specimen of the plate VCI_UD_3 (sample area A). A local reference area has been defined conservatively with a size of 1 mm^2^. The areas of the identified voids are determined and set in relation to the reference area (see Figure 6). The measurement results in a local void content of 1.88%. The result is higher than the overall measured value of −0.66%. The result shows that the overall and the local void content is lower than 2%, which is in the range of a high-performance laminate [26].

In Figure 7, representative cross-sections are displayed for each plate with a 0/90° layer configuration. For both processes, very homogeneous laminates are formed, whereas the individual layers remain recognizable. Due to the presence of binder and the consolidation pressure of 1 bar, the boundaries between the individual layers are presumably not blurred. Only very few voids are observed, which correlates with the measured void contents. Small undulation and resin-rich zones only appear next to the gaps that have been introduced in the preforms during the laying process. The VAP plates show small thickness deviations since there was only a one-sided mold (glass plate). Compared to the unidirectional VAP plates, the surface—specifically, the first and last layer—does not show an undulation effect from the used peel ply or flow medium. For both processes, the detected voids are almost round-shaped, having a typical size of 25–50 µm in diameter. Single larger voids are oval-shaped and can show lengths up to 150 µm with a height of 50 µm. They mainly appear in between the tows. On single spots, voids have been detected inside of the tow having diameter sizes of 25 µm and smaller.

### 3.2. Mechanical Properties

In Table 7, all measured mechanical properties are displayed. The values are given absolutely and normalized to 40% FVF. All standard deviations are in a typical range for the type of testing method. Therefore, all values can be regarded as reliable and representative. For the normalized values, the standard deviation is spared since a scaling of this value is not plausible and the normalization of the mechanical value itself is done under the estimation that the values have quasilinear behavior within a small range around the measured FVF. The absolute tensile values for the VCI process are lower than for the VAP reference. The absolute bending values are higher for the VCI. The normalized values show that the potential of the VCI process is similar to the VAP process. The normalized values are higher for VCI than for VAP, except for the tensile values for 0/90°. The range is between 18% for the bending modulus 0/90° up to 26% for the tensile modulus 0°. The normalized VCI value for tensile strength 0/90° and tensile elongation 0/90° is 13%, respectively, 3.5% lower than for VAP. The absolute elongation values are all lower for the VCI process.

### 3.3. Consumables and Process Time Infusion

Table 8 shows the accumulated consumable materials, and the labor time required to manufacture a single plate with VCI and VAP. The results show that there is a significantly higher demand for a wide variety of materials for the VAP process. The VCI only requires two multi-layer bags, and the outer bag can be reused. The used bags are self-releasing so there is no release agent/wax needed. The time for setting up and performing the infusion is four times less for the VCI compared to VAP. The time value does not include resin preparation and curing since these values are similar for both processes. The infusion time is the same for both processes.

## 4. Discussion

The results show that using the vacuum chamber infusion process results in similar laminate qualities as the VAP process when using the same dry fiber placement preform layup. In particular, the low void content of less than 2% and fiber undulation indicate a good industrial application in the future for the new process, although it was recognized that there was a slight fiber washing for the unidirectional preform due to a missing compaction pressure during the infusion process. No fiber washing was observed in the 0/90° oriented fibers because the perpendicular orientation keeps the tows in place.

Values between 39% and 45% are observed for the fiber volume fraction for both infusion methods, while the values for the VCI method are in the lower range. The authors estimate that the generally low FVF depends on the preform and not on the process itself. The reason is seen in the used DFP preform, which has a binder content of 8% and a fiber spacing of 2 mm when deposited. [27] reports a reduction in fiber content for DFP laminates with a 0.8 mm gap of 5% in fiber volume fraction compared to a reference laminate without gaps. Studies with other semi-finished products have shown that fiber volume contents beyond 52% are otherwise reachable with the VAP process [16,28]. A possible reason for the low FVF for VCI may be that the amount of resin used was too high. Initially, the amount was chosen to ensure a safe complete impregnation without visible dry spots on the plate surfaces. Since this process is being analyzed for the first time, the amount used and its position are not yet optimized. If the resin distribution inside the bag is optimized, it offers the chance to find an optimum for good impregnation, thereby increasing the FVF. Additionally, the VCI also offers the option of consolidating the impregnated preform by using a press to further increase the FVF. The preform can be kept inside the bag, and placed in the press, and excessive resin can be removed and stay inside the bag. However, it should be taken into consideration that using a press would increase the complexity and diminish some advantages of the VCI in the way described in Section 2.1. As 39% FVF is too low for high-performance composite applications, the VCI should be further optimized. Studies with unidirectional and multi-axial non-crimp fabrics have to be performed to evaluate the maximum achievable FVF and investigate the void formation and fiber washing for these types of preforms. Additionally, alternative ways of consolidation have to be investigated.

Furthermore, the mechanical properties measured suggest great potential for structural applications in both processes. When the absolute values are normalized to a common fiber volume content of 40%, the VCI values for tensile 0° and bending 0/90° are approx. 10% higher than the measured characteristic values for VAP. The higher normalized values for VCI tensile 0° can be explained by the minor laminate quality of the unidirectional VAP plates, where surface unevenness, high thickness variation, and undulations perpendicular to the 0° fiber direction are overserved in the cross-sections.

The normalized value for the tensile 0/90° configuration shows 26% higher strength values for the VAP process. The stiffness is 4% higher, though it is in the range of the standard deviation. The normalized 0/90° bending value for strength is 11% higher and for stiffness 16% higher for the VCI process. Since the 0/90° laminates show a higher homogeneity for both processes, which is revealed by the microscopic analysis, these values can be regarded as more reliable for comparison and evaluation of the process potential. Since the difference in the result of the two values for bending and tensile is minimal and the differences are within the range of the standard deviations, a similar quality regarding the mechanical properties can be stated for both processes.

Due to this fact and the overall low void content, a correlation between the absolute void content and the mechanical properties of the 0/90° laminates cannot be worked out. Regarding the values of the unidirectional laminates, it can be clearly stated that the bad surface condition of the VAP plates lowered the tensile strength compared to the VCI values. The induced undulation resulting from the resin flow for VCI and the undulation introduced into the VAP laminates by the release fabric results in lower mechanical values than are theoretically possible. For a unidirectional laminate, the theoretical maximums for the VCI and VAP are displayed in Table 9 with respect to the measured FVF. The calculation is performed according to [29]:

(2) $$E_{matrix}\ll E_{fiber}$$

(3) $$E_{UD\;laminate}\approx \;E_{fiber}*FVF$$

It can be seen that the reached tensile strength value for VAP is with 25% lower than it is for VCI, with 19%. The theoretical maximum for the stiffness is reached for VCI, whereas the value for VAP is 10% lower. By using an optimized VAP infusion setup, such as adding a caul plate or using a different flow medium, this issue could be addressed. The significant economic finding was demonstrated that VCI requires fewer consumables, and its setup is four times faster compared to VAP. The infusion time was similar because the pressure difference was the same, and the preform was identical. This results in reduced production costs, higher unit output, and lower resource consumption. When vacuum bagging, double the amount in VCI is needed because the entire preform is wrapped in an inner bag. The outer bag can be reused since it does not come into contact with resin, making it easier to recycle and reuse. As a result, the VCI process has a lower environmental impact due to the overall reduction of consumables and the use of only one material type, which makes it easier to recycle.

At the current stage of development, the low fiber volume fraction of 39% is limiting potential applications. The following applications could be possible when only considering the restrictions of the dimensions of the used chamber: for the automotive and aerospace sectors, flat or slightly curved plate structures, possibly with stiffening beads, and simple 2D components such as deflection levers or brackets; in the sports sector, parts such as skateboards, spring elements in shoe insoles, or rackets could be considered. There is also great potential in the field of medical technology, with possible applications including orthoses/prostheses, parts of walking aids, or wheelchairs. In this area, in particular, a low-cost manufacturing process offers high social added value, as it would give more people access to this technology [30]. To address the issue of size, a test setup using a larger vacuum chamber has to be conducted. Larger vacuum chambers already exist on the market and can be custom-made. An economic analysis needs to be conducted to determine the chamber size at which the cost of building and operating the chamber outweighs the benefits. Additionally, there are expected scaling issues that will need to be addressed. These include handling the preform, any required molds, and the resin system.

## 5. Conclusions and Prospect

The scope of this work was to analyze if similar results in mechanical properties and laminate quality of the proposed VCI process in comparison to the state-of-the-art VAP process can be performed and which economic benefits can be achieved. The manufacturing approach has been thoroughly investigated for the first time, and the key findings are as follows:

1. The DFP laminates produced using the VCI process exhibit comparable mechanical properties to those produced using the VAP process when normalized to a similar fiber volume content. The absolute values are in a relevant range for industry applications. The normalized values for the tensile strength and modulus of 0/90° configurations are 772 MPa and 52 GPa for VCI and 975 MPa and 54 GPa for VAP. For the bending strength and stiffness, the size ratio of the values is reversed, with higher values for VCI with 646 MPa and 50 GPa and 851 MPa and 43 GPa for VAP.
2. The fiber volume fraction for VCI with 39.0% is about six percentage points below the value of VAP. Process optimization has not been performed yet, allowing room for improvement, especially with non-bindered preforms like non-crimped fabrics.
3. It is shown that the laminate quality of the infusion process of a one-time evacuated and then sealed system (closed system) in terms of void content is equal to an infusion process of a permanently evacuated setup (open system). The void content is below 2% for both the VCI and VAP processes. This result makes it suitable for industrial use [26]. The low void contents for VAP have been confirmed.
4. The number of different consumable materials required for VAP can be significantly reduced with VCI, resulting in a drastic 4× timesaving in the preparation of the infusion process compared to the VAP method.

However, for good impregnation, the compatibility between resin viscosity and preform permeability is essential and is also a challenge within the vacuum chamber process. For the VCI, this is even more so, since there is no permanent evacuation. The limitations are predicted to be in the number of layers respectively the thickness of the preforms depending on their permeability. The limitations are predicted to be in the number of layers, with the thickness of the preforms depending on their permeability. Therefore, process optimizing studies have to be performed to analyze the infusion behavior more in detail, increasing FVF by using different dry preforms like fabrics or non-crimp fabrics. Despite having flat plates of 320 mm × 330 mm in size, no complex 3D geometries were manufactured using VCI. These geometries need to be approved in future studies. To achieve more complex geometries, two approaches are possible, and both are recommended for further study:

1. The final contoured preform is only impregnated using the VCI process. The infusion structure can then be molded and cured in a consolidation tool. It is also possible to remove the impregnated preform from the bag and then place it in a consolidation mold, like using a standard prepreg material.
2. The VCI process can also be carried out in a sufficiently rigid thin-walled mold. This mold can, e.g., be produced by injection molding or thermoforming from a thermoplastic material. Thin metal sheets are also conceivable.

The limitations of the VCI process arise from the size of the available vacuum chamber, the forming of three-dimensional infusion bags, and the thermoformed molds. Large vacuum chambers measuring 5.5 m in diameter and 12 m in length are available [31] and thermoforming parts with sizes up to 4 m^2^ [32] are feasible, defining the potential dimensions. It is important to validate the feasibility of implementing geometric upscaling to increase its industrial appeal. In addition, VCI offers the highest potential for automation among all open mold LRI methods, as it requires only one consumable material. It should be noted that the original vacuum chamber process in food packaging is already highly automated [33]. With this major advantage, the authors see a very large scaling potential with VCI in terms of the number of units that can be produced.

## Figures and Tables

**Table 1:** Used materials.

	Description
Fiber	Mitsubishi 30 k GRAFIL TRH50 30 K 0.9%R 8.00KGS ML CP (Mitsubishi Chemical Europe GmbH, Düsseldorf, Germany)
Binder	Hexion Epikote Resin TRAC 06720-3 (Hexion Inc., Columbus, OH, USA)
Matrix	Hexion RIMR135/RIMH137 (Hexion Inc., Columbus, OH, USA)
Degasser	BYK-A 530 (BYK-Chemie GmbH, Wesel, Germany)
Substrate	Hexcel, HexForce 02116 1260 TF970, 105 g/m^2^, plain weave (Hexcel Corporation, Stamford, CT, USA)

**Table 2:** FixedTow properties.

FixedTow	Unit	Value
Width	[mm]	20
Binder, one-sided	[mass-%]	8
Tex fiber	[tex]	1677

**Table 3:** Properties of the fiber.

Fiber Mitsubishi	Unit	Value
Strength	[GPa]	5.51
Modulus	[GPa]	260
Density	[g/cm^3^]	1.82
Yield	[tex]	1677
Size content	[mass-%]	0.9

**Table 4:** Layer setup for the preforms.

Configuration	Layers	Orientation
UD	2 × 6	[0°/0°/0°/0°/0°/0°] s
0°/90°	4 × 6	[[0°/90°/0/90°/0°/90°] + [90°/0°/90°/0°/90°/0°]] s

**Table 5:** Configuration for the infusion and mechanical testing methods.

Configuration				Testing
Process	Fiber Orientation	Layer Amount	Plate Thickness	Standard *
VAP	0°	12	1 mm	Tensile
VCI	0°	12	1 mm	
VAP	0°/90°	24	2 mm	Tensile
VCI	0°/90°	24	2 mm	Bending

* DIN-EN ISO 527-5, DIN-EN ISO 527-4, DIN-EN ISO 14125

**Table 6:** Results for FVF, void content, and density measurements. All values are average values from all three sample places, A, B, and C.

	Fiber Volume Fraction	Void Content *	Density
	[%]	[%]	[g/cm^3^]
VCI_UD_1	34.9 (±1.7)	−0.66 (±0.35)	1.38 (±0.01)
VCI_UD_2	38.7 (±1.4)	0.02 (±0.61)	1.40 (±0.01)
VCI_UD_3	39.3 (±0.9)	−0.66 (±0.35)	1.41 (±0.01)
VCI_0_90_1	42.9 (±1.0)	1.15 (±0.92)	1.42 (±0.01)
VCI_0_90_2	40.5 (±1.3)	−0.05 (±0.43)	1.42 (±0.01)
VCI_0_90_3	37.6 (±0.5)	−0.62 (±0.57)	1.40 (±0.01)
VAP_UD_1	42.7 (±1.0)	2.89 (±0.56)	1.39 (±0.01)
VAP_UD_2	48.5 (±1.0)	1.61 (±0.62)	1.45 (±0.01)
VAP_UD_3	48.5 (±0.8)	0.94 (±0.45)	1.45 (±0.01)
VAP_0_90_1	39.5 (±2.2)	0.20 (±0.30)	1.40 (±0.01)
VAP_0_90_2	44.0 (±1.0)	1.06 (±0.45)	1.43 (±0.01)
VAP_0_90_3	47.8 (±1.5)	1.79 (±1.71)	1.44 (±0.01)
Average VCI	39.0 (±2.7)	−0.2 (±0.70)	1.41 (±0.01)
Average VAP	45.2 (±3.7)	1.41 (±0.92)	1.43 (±0.02)

* Negative values are physically not possible; the explanation can be found in the text.

**Table 7:** Results of the mechanical testing, tensile, and bending test.

Property	Unit	Absolute Value *		Normalized Value **	
		VCI	VAP	VCI	VAP
Tensile strength 0°	[MPa]	1750 (±127)	1871 (±68)	1865	1609
Tensile modulus 0°	[GPa]	101 (±6)	105 (±4)	108	91
Tensile elongation 0°	[%]	1.55 (±0.06)	1.67 (±0.06)	1.65	1.43
Tensile strength 0/90°	[MPa]	779 (±60)	1058 (±58)	772	975
Tensile modulus 0/90°	[GPa]	52 (±2)	59 (±2)	52	54
Tensile Elongation 0/90°	[%]	1.39 (±0.06)	1.65 (±0.06)	1.38	1.52
Bending strength 0/90°	[MPa]	674 (±29)	631 (±45)	646	581
Bending modulus 0/90°	[GPa]	52 (±2)	47 (±3)	50	43
Elongation edge fiber 0/90°	[%]	1.42 (±0.06)	1.48 (±0.06)	1.37	1.35

* The values are the average values of all 3 tested plates for each configuration. ** Normalized to 40% FVF. For normalization, the FVF of the single plates was used and, then, the average value was determined.

**Table 8:** Used consumables materials for 1 plate comparison VCI vs. VAP and time.

VCI		VAP	
**Consumables**			
Material	Dimensions	Material	Dimensions/Weight
PA/PE bag 90 µm	2 × 400 mm × 600 mm	Tacky Tape (Butyl)	2.5 m
		Vacuum bag (PA)	400 mm × 600 mm
		VAP Membrane	380 mm × 580 mm
		Peel ply (PA6)	2 × 300 mm × 300 mm
		Tube (PE) 8 mm	1 m
		Flow mesh (PET)	300 mm × 300 mm
		Adhesive tape (Acryl)	10 mm × 20 cm
		Omega profile (Silicone)	25 cm
		Release wax	5 g
**The time needed to set up and perform the infusion of a 1–2 mm plate 320 mm × 330 mm**			
Preparation	7 min	52 min	
Infusion	8 min	8 min	
Total	15 min	60 min	

**Table 9:** The theoretical maximum of unidirectional tensile strength/stiffness compared to the measured value.

		Fiber	VCI	VAP
FVF	[%]	100	39	45
Strength max	[MPa]	5510	2149	2479
Strength measured	[MPa]	---	1750	1871
**Difference**	**[%]**	**---**	**19**	**25**
Stiffness max	[GPa]	260	101	117
Stiffness measured	[GPa]	---	101	105
**Difference**	**[%]**	**---**	**0**	**10**

## Data Availability

Material support VAP membrane type CS/E from Composyst GmbH, Landsberg am Lech, Germany.
